# Predictive value of peripheral blood CD4^+^ T and NK cells on efficacy and long-term survival in advanced HCC patients receiving immunotherapy

**DOI:** 10.3389/fimmu.2025.1683328

**Published:** 2025-11-28

**Authors:** Zheng Pan, Shihang Song, Yuan An, Lianyue Guan, Hongyu Liu, Wei Li

**Affiliations:** 1 Department of Hepatobiliary-Pancreatic Surgery, China-Japan Union Hospital of Jilin University, Changchun, China; 2 Department of Thoracic and Cardiovascular Surgery, Ezhou Central Hospital, Ezhou, China

**Keywords:** CD4+ T cells, NK cells, hepatocellular carcinoma (HCC), immunotherapy, treatment efficacy, overall survival (OS), progression-free survival (PFS)

## Abstract

**Background:**

Early assessment of the efficacy of immune checkpoint inhibitor (ICI) therapy in advanced hepatocellular carcinoma (HCC) patients plays a crucial role. In this study, we systematically analyzed the predictive values of various lymphocyte subpopulations (CD4^+^ T, CD8^+^ T, and NK cells) at different time points of pretreatment (W0), 3 weeks (W3) and 6 weeks (W6) post-treatment and their relative changes compared to W0 for objective response (OR).

**Materials and methods:**

We systematically reviewed the clinical data of these patients in our hospital. Logistic regression analyses were conducted to identify variables predicting OR. Cut-off values were determined by maximizing Youden index. Then, patients were stratified based on these cut-off values, and Kaplan-Meier analysis with log-rank test was used to compare progression-free survival (PFS) and overall survival (OS) between groups.

**Results:**

A total of 41 patients were included. Multivariate logistic regression analysis revealed that W3 CD4^+^ T cells (%) and W3 NK cells(%) were independent predictors of treatment efficacy. The odds ratios (95% confidence interval) and p-values were 1.140 (1.020-1.274), 0.021 and 2.232 (1.025-4.860), 0.043, respectively. Combining these two indicators yielded even better performance: all patients in the “All High” group achieved OR, whereas none in the “All Low” group did. Furthermore, whether used individually or in combination, they all could stratify patients by PFS and OS.

**Conclusions:**

W3 CD4^+^ T cells (%) and W3 NK cells (%) can predict the efficacy and long-term survival (PFS and OS) of these patients. Their combination offers improved predictive performance.

## 1 Introduction

Liver cancer poses a significant threat to human health. In 2022, there were estimated 865,000 new cases and 757,000 deaths globally, resulting in a high mortality rate up to 88% (1). Additionally, liver cancer ranks as the 6th most common malignancy worldwide and is the 3rd leading cause of cancer-related deaths (1). Approximately 80% of primary liver cancer are hepatocellular carcinoma (HCC) (2), which is the central topic of this article. Most HCC patients are already in the advanced stage at their initial diagnosis. For instance, about 64% of HCC patients in China are diagnosed at an intermediate to advanced stage (3). Previously, treatment options for these patients were limited, and their prognosis was extremely poor. Recently, novel immunotherapies, known as immune check point inhibitor (ICI), including PD-1, PD-L1, and CTLA-4 inhibitors, have shown encouraging efficacy in patients with advanced HCC. The IMbrave150 study demonstrated that the combination of atezolizumab (one kind of PD-L1 inhibitor) and bevacizumab was superior to sorafenib monotherapy, significantly improving patients’ OS (12-month OS rate: 67.2% *vs*. 54.6%) and PFS (median: 6.8 months *vs*. 4.3 months) (4). Nevertheless, due to the tumor heterogeneity, patients exhibit significant variations in response to immunotherapy, with objective response rates (ORR) ranging only from 15% to 32% (5). Therefore, there’s an urgent need for a precise and convenient biomarker that can accurately predict the efficacy and long-term survival, including overall survival (OS) and progression-free survival (PFS), of patients with advanced HCC undergoing ICI immunotherapy.

Immunotherapy for HCC works by blocking overactive immune checkpoints in the tumor microenvironment, thereby restoring anti-tumor immunity (6, 7). Lymphocytes play a central role in this anti-tumor immune response. Previous studies have shown that elevated intratumoral CD8^+^ T cells are associated with improved prognosis (8). However, assessing intratumoral lymphocytes status relies on tissue biopsy, which is invasive and may lead to needle tract seeding (9).

An increasing number of researches have shifted their attention to peripheral blood lymphocytes. Previous studies have shown that the level of specific lymphocyte subpopulations before treatment, or their dynamic changes during treatment, are associated with treatment efficacy and long-term survival in patients with advanced HCC receiving specific immunotherapy (10–12). However, the immune system is highly dynamic. With the advancement of immunotherapy, the immune system will respond accordingly, leading to changes in these lymphocyte subpopulations. Therefore, the percentage of lymphocyte subpopulations at different time points after ICI treatment may possess predictive value for treatment efficacy and long-term survival. In this study, we collected the percentages of peripheral blood lymphocyte subpopulations at the time points of pretreatment (W0), 3 weeks (W3) and 6 weeks (W6) after treatment from patients with advanced HCC receiving ICI immunotherapy in our hospital. Then, the predictive values of these lymphocyte subpopulations at different time points and their relative changes compared to W0 for treatment efficacy and long-term survival, including PFS and OS, were systematically analyze. The goal is to identify a precise, convenient, noninvasive, and reliable biomarker for the prediction of therapeutic efficacy and long-term survival in patients receiving ICI immunotherapy, supporting individualized treatment strategies in clinical practice.

## 2 Materials and methods

### 2.1 Patients and data collection

Clinical data of patients with advanced HCC who received ICI immunotherapy at our hospital between September 2020 and December 2021 were collected retrospectively. This study was approved by the Institutional Review Board of China-Japan Union Hospital of Jilin University (2025110501). Informed consent was waived due to the nature of retrospective studies. The inclusion and exclusion criteria were as follows:

Inclusion criteria: 1. Diagnosed with HCC according to the Chinese Guidelines for the Diagnosis and Treatment of Primary Liver Cancer (2022 Edition) (13), with a clinical stage of IIIa or higher, which is unresectable, at initial diagnosis; 2. Availability of complete laboratory test results and follow-up data.

Exclusion criteria: 1. Underwent surgery within the past; 2. Recent history of severe inflammatory diseases; 3. Recent use of corticosteroids, immunosuppressants, or similar medications; 4. Presence of other types of malignancies; 5. Coexisting hematologic malignancies; 6. Missing substantial clinical data or poor data integrity.

The ICI used in this study included Toripalimab (15, 36.5%), Camrelizumab (9, 21.9%), Sintilimab (14, 34.1%), and Tislelizumab (3, 7.3%), all administered intravenously at a dose of 200mg every 3 weeks. Treatment was continued until disease progression, unacceptable toxicity, death, or any other reasons.

The median treatment cycle of all patients is 7.6 cycles. Adverse reactions were observed in some patients, including rash (11, 26.8%), fatigue (8, 19.5%), hypertension (7, 17.1%), paresthesia (6, 14.6%), and nausea (6, 14.6%).

In addition to routine clinical data, we specifically collected the percentages of peripheral blood lymphocyte subpopulations before treatment (W0), as well as 3 weeks (W3) and 6 weeks (W6) after treatment. The lymphocyte subpopulations included CD4^+^ T cells, CD8^+^ T cells, and NK cells. Peripheral blood samples were collected from patients and analyzed by flow cytometry in the Department of Laboratory Medicine at our hospital.

The relative change rates were defined as follows: ΔW3 = (W3 value - W0 value)/W0 value × 100%; ΔW6 = (W6 value - W0 value)/W0 value × 100%.

### 2.2 Efficacy evaluation and long-term follow-up

After initiating immunotherapy, patients underwent regular contrast-enhanced CT or MRI scans to evaluate treatment response, according to the modified Response Evaluation Criteria in Solid Tumors (mRECIST) (14). The optimal response achieved during the therapy was recorded. Objective response (OR) was defined as complete response (CR) or partial response (PR), while non-objective response (NOR) included stable disease (SD) and progressive disease (PD).

Long-term survival was obtained through regular telephone follow-ups, with the last follow-up performed in June 2025. OS was defined from the first administration of PD-1 inhibitor to death for any cause or the date of last follow-up. PFS was defined as the time from the initiation PD-1 inhibitor therapy to either progression or death for any cause.

### 2.3 Cell detection

The cell analysis was performed by flow cytometer (Model FC 500, Beckman Coulter, USA), and all reagents were manufactured by Beckman Coulter, USA. 1 ml of peripheral blood was collected from the patient and transferred into an EDTA-anticoagulated vacuum tube, followed by thorough mixing to prevent coagulation. 2 flow cytometry sample tubes were prepared and labeled as T cells tube and NK cells tube, respectively. To each tube, 100 μl of anticoagulated whole blood sample was added, followed by the addition of 20 μl of T lymphocyte subset antibody (CD45-FITC/CD4-PE/CD8-ECD/CD3-PC5, Cat. No.: 6607013) and NK cell antibody (CD45-FITC/CD56-PE/CD19-ECD/CD3-PC5, Cat. No.: 6607073), respectively. After thorough mixing, the tubes were incubated for 20 minutes at room temperature in the dark. Then, 500 μl of red blood cell lysis solution (OptiLyse B No-Wash Lysing Solution, Cat. No.: IM1400) was added to each tube. Following mixing, the tubes were incubated for 20 minutes at room temperature protected from light. Subsequently, 500 μl of phosphate-buffered saline (PBS) was added, and the samples were mixed and centrifuged at 1,000 r/min for 5 minutes. The supernatant was discarded, and the washing step was repeated once under the same conditions. Finally, after removal of the supernatant, 500 μl of PBS was added to each tube, and the samples were analyzed using flow cytometer. The CD4^+^ T cells were identified as CD45^+^ CD3^+^ CD4^+^ CD8^−^, CD8^+^ T cells were CD45^+^ CD3^+^ CD8^+^ CD4^−^, and NK cells was CD45^+^ CD3^−^ CD19^−^ CD56^+^.

### 2.4 Statistical analysis

The Shapiro-Wilk test was used firstly to assess the normality of continuous variables. Continuous variables with a normal distribution were described as mean ± standard deviation (SD), while non-normally distributed variables were presented as median with interquartile range (IQR). Categorical variables were described as counts and percentages.

In order to compare differences between the OR and NOR, independent samples t-tests were used for normally distributed continuous variables, while Mann-Whitney U tests were applied for non-normally distributed ones. Categorical variables were compared using the Chi-square test when the expected frequency in each cell was ≥5; otherwise, Fisher’s exact test was employed. Variables with p < 0.1 were included in the univariate logistic regression. Those with p < 0.1 in the univariate logistic regression were further subjected to stepwise selection, followed by multivariate logistic regression to identify independent predictors of OR. The predictive performance of selected variables was assessed using receiver operating characteristic (ROC) curve, and the optimal cut-off values were determined based on maximizing Youden index. The decision curve analyses (DCA) were utilized to evaluate the net benefit of different lymphocyte subpopulations (15). Bootstrap internal validation was used to evaluate the stability and generalization of the model. Kaplan-Meier survival curves with log-rank tests were used to compare PFS and OS across groups. Since this was a retrospective exploratory analysis, the sample size was determined by the available eligible patients during the study period, and no *a priori* sample size calculation was performed. All statistical analyses were performed using R (version 4.4.1) and Python (version 3.12.3). The p-value < 0.05 was considered statistically significant.

## 3 Results

### 3.1 Baseline characteristics of all patients

A total of 41 patients were included. There were 21 patients (51.22%) who were aged 60 years or older. The majority patients were male (30 patients, 73.17%). Most of the patients were HBsAg-positive (33 patients, 80.49%), stage III according to the Barcelona Clinic Liver Cancer Staging System (BCLC) staging system (16) (31 patients, 75.61%), Child–Pugh grade A (33 patients, 80.49%). 17 patients (41.46%) had an alpha-fetoprotein (AFP) level ≥ 400 ng/ml, and 18 patients (43.90%) had a des-gamma-carboxy prothrombin (DCP) level ≥ 40 ng/ml. Regarding to the treatment response, 13 patients (31.71%) achieved PR, 15 patients (36.59%) had SD, and 13 patients (31.71%) experienced PD; no patients achieved CR. A total of 13 patients (31.71%) were OR, while 28 patients (68.29%) were NOR (**Table 1a**). The details regarding lymphocyte-related values are presented in **Table 1b**.

**Table 1a:** Baseline characteristics of all patients.

Variables		n=41
Age, years	≥60	21 (51.22%)
	<60	20 (48.78%)
Sex	Male	30 (73.17%)
	Female	11 (26.83%)
HBsAg	Positive	33 (80.49%)
	Negative	8 (19.51%)
BCLC stage	III	31 (75.61%)
	IV	10 (24.39%)
Child-Pugh grade	A	33 (80.49%)
	B	8 (19.51%)
ALT (U/L)		32.10 (26.10, 45.60)
AST (U/L)		29.50 (22.50, 39.00)
AFP (ng/ml)	<400	24 (58.54%)
	≥400	17 (41.46%)
Response evaluation	PR	13 (31.71%)
	SD	15 (36.59%)
	PD	13 (31.71%)

**Table 1b:** Clinical characteristics of all patients regarding lymphocyte.

Variables		n=41
W0	WBC (10^9^/L)	7.92 ± 2.09
	Lym (10^9^/L)	1.68 ± 0.31
	CD4^+^ T cells (%)	32.70 (25.93, 42.12)
	CD8^+^ T cells (%)	23.60 (20.20, 26.20)
	NK cells (%)	10.97 ± 4.52
	CD4^+^ T/CD8^+^ T cells	1.35 (1.04, 1.79)
W3	WBC (10^9^/L)	7.32 (5.63, 11.35)
	Lym (10^9^/L)	2.14 (1.59, 2.48)
	CD4^+^ T cells (%)	37.15 (28.84, 41.71)
	CD8^+^ T cells (%)	24.40 (21.30, 26.60)
	NK cells (%)	9.10 (5.90, 12.60)
	CD4^+^ T/CD8^+^ T cells	1.45 (1.07, 1.69)
W6	WBC (10^9^/L)	7.99 ± 2.10
	Lym (10^9^/L)	1.95 ± 0.36
	CD4^+^ T cells (%)	33.00 (27.98, 45.02)
	CD8^+^ T cells (%)	23.10 (20.10, 25.80)
	NK cells (%)	10.94 ± 4.42
	CD4^+^ T/CD8^+^ T cell	1.46 (1.20, 1.67)

Continuous variables were expressed as mean ± standard deviation if normally distributed, or as median (interquartile range) if not. Categorical variables were presented as counts and percentages.

HBsAg, surface antigen of the hepatitis B virus; BCLC, Barcelona Clinic Liver Cancer Staging System; ALT, alanine aminotransferase; AST, aspartate aminotransferase; AFP, alpha-fetoprotein; DCP, des-gamma-carboxyprothrombin; WBC, white blood cell; Lym, lymphocyte; PR, partial response; SD, stable disease; PD, progressive disease; W0, pretreatment; W3, 3 weeks post-treatment; W6, 6weeks post-treatment.

### 3.2 Comparison between OR and NOR

Statistical analysis revealed no significant differences in baseline characteristics between OR and NOR, including age, sex, HBsAg, BCLC stage, Child-Pugh grade, AFP, and DCP (**Table 2a**).

**Table 2a:** Baseline characteristics comparison between objective response (OR) and non-objective response (NOR) patients.

Variables		OR (13)	NOR (28)	p value
Age, years	<60	7 (53.8%)	15 (53.6%)	0.915
	≥60	6 (46.2%)	13 (46.4%)	
Sex	Male	10 (76.9%)	20 (71.4%)	1.000
	Female	3 (23.1%)	8 (28.6%)	
HBsAg	Positive	10 (76.9%)	23 (82.1%)	0.693
	Negative	3 (23.1%)	5 (17.9%)	
BCLC stage	III	12 (92.3%)	19 (67.9%)	0.129
	IV	1 (7.7%)	9 (32.1%)	
Child-Pugh grade	A	11 (84.6%)	22 (78.6%)	1.000
	B	2 (15.4%)	6 (21.4%)	
AFP (ng/ml)	<400	9 (69.2%)	15 (53.6%)	0.499
	≥400	4 (30.8%)	13 (46.4%)	
DCP (ng/ml)	<40	9 (69.2%)	14 (50.0%)	0.321
	≥40	4 (30.8%)	14 (50.0%)	
ALT (U/L)		39.36 ± 21.07	32.60 (25.98, 45.23)	0.978
AST (U/L)		27.40 (22.80, 36.50)	29.65 (22.38, 40.92)	0.779

However, several lymphocyte-related variables demonstrated statistically significant differences between the two groups. Variables with p < 0.1 are highlighted in bold in **Table 2b**.

**Table 2b:** Clinical characteristics regarding lymphocytes comparison between objective response (OR) and non-objective response (NOR) patients.

Variables		OR (13)	NOR (28)	p value
W0	WBC (10^9^/L) Lym (10^9^/L)	8.04 ± 1.79 1.76 ± 0.16	7.86 ± 2.24 1.64 ± 0.36	0.790 0.147
	**CD4^+^ T cells (%)**	42.71 ± 18.33	32.51 ± 10.80	0.081
	CD8^+^ T cells (%)	23.72 ± 4.22	23.35 (20.18, 25.73)	0.449
	**NK cells (%)**	12.85 ± 4.8	10.10 ± 4.18	0.092
	**CD4^+^ T/CD8^+^ T cells**	1.88 ± 0.89	1.40 ± 0.47	0.085
W3	WBC (10^9^/L) Lym (10^9^/L)	9.26 ± 3.30 2.03 ± 0.49	7.29 (5.61, 10.69) 2.18 (1.58, 2.50)	0.293 1.000
	**CD4^+^ T cells (%)**	40.61 (37.57, 70.91)	32.42 ± 10.41	0.001
	CD8^+^ T cells (%)	24.35 ± 3.78	24.25 (21.05, 26.45)	0.624
	**NK cells (%)**	15.22 ± 6.19	7.67 ± 2.82	0.001
	**CD4^+^ T/CD8^+^ T cells**	2.23 ± 1.01	1.27 (1.06, 1.58)	0.006
W6	**WBC (10^9^/L)** Lym (10^9^/L)	9.56 ± 1.72 2.00 ± 0.31	7.27 ± 1.86 1.93 ± 0.39	0.001 0.557
	**CD4^+^ T cells (%)**	43.53 ± 16.96	34.56 ± 10.70	0.098
	**CD8^+^ T cells (%)**	21.23 ± 3.44	25.35 ± 6.16	0.009
	**NK cells (%)**	13.02 ± 4.81	9.97 ± 3.95	0.060
	**CD4^+^ T/CD8^+^ T cells**	2.12 ± 0.95	1.32 (1.15, 1.56)	0.006
ΔW3 (%)	WBC Lym	17.47 ± 38.20 15.40 ± 26.76	7.61 ± 45.08 29.38 ± 43.61	0.474 0.216
	**CD4^+^ T cells**	30.03 ± 44.08	-3.74 (-14.29, 16.17)	0.055
	CD8^+^ T cells	3.43 ± 8.26	3.00 ± 7.41	0.874
	**NK cells**	21.43 ± 25.40	-23.65 (-34.35, -13.72)	0.0001
ΔW6 (%)	WBC Lym	26.86 ± 43.70 13.45 ± 13.72	-2.93 ± 26.47 24.21 ± 40.91	0.037 0.220
	CD4^+^ T cells	5.77 ± 28.19	13.06 ± 35.74	0.486
	**CD8^+^ T cells**	-9.84 ± 9.20	2.76 (-7.11, 31.43)	0.006
	NK cells	7.36 ± 36.85	-4.75 (-17.28, 19.80)	0.834
ΔW3 (%)/ΔW6 (%)	WBC	0.70 ± 1.61	0.59 (-1.41, 1.65)	0.705
	Lym	1.07 (0.43, 2.23)	0.99 (0.21, 1.55)	0.624
	CD4^+^ T cells	0.59 (-0.09, 2.12)	0.45 (0.08, 1.13)	0.475
	CD8^+^ T cells	-0.39 (-1.12, 0.12)	-0.16 (-0.81, 0.24)	0.566
	NK cells	0.31 ± 1.30	0.65 (-0.26, 1.77)	0.493

Continuous variables were expressed as mean ± standard deviation if normally distributed, or as median (interquartile range) if not. Categorical variables were presented as counts and percentages. Variables with p < 0.1 are highlighted in bold.

HBsAg, surface antigen of the hepatitis B virus; BCLC, Barcelona Clinic Liver Cancer Staging System; ALT, alanine aminotransferase; AST, aspartate aminotransferase; AFP, alpha-fetoprotein; DCP, des-gamma-carboxyprothrombin; WBC, white blood cell; Lym, lymphocyte; W0, pretreatment; W3, 3 weeks post-treatment; W6, 6 weeks post-treatment; ΔW3=(W3-W0)/W0*100%; ΔW6=(W6-W0)/W0*100%.

The variables regarding lymphocytes absolute counts were also analyzed. Detailed statistical results are provided in the **Supplementary Materials** (**Supplementary Tables S1**, **S2**). Unfortunately, neither the absolute counts nor the derived indexes demonstrated predictive efficacy.

### 3.3 Prediction for OR

Variables with p < 0.1 from the comparative analysis in section 3.2 were included in a univariate logistic regression analysis (**Table 3a**). Variables with p < 0.1 in the univariate analysis were then subjected to stepwise selection and multivariate logistic regression (**Table 3b**). CD4^+^ T cells (%) and NK cells (%) at W3 were identified as independent predictors of OR. The odds ratios [95% confidence intervals, 95% CI] were 1.140 [1.020-1.274] and 2.232 [1.025-4.860], with p-values of 0.021 and 0.043, respectively.

**Table 3a:** Univariate logistic regression of lymphocyte subpopulations percentage to predict objective response.

Variables		Odds ratio (95% Confidence Interval)	p value
W0	CD4^+^ T cells (%)	1.056 (1.000-1.116)	0.051
	NK cells (%)	1.153 (0.984–1.350)	0.079
	CD4^+^ T/CD8^+^ T cells	3.172 (1.036–9.709)	0.043
W3	CD4^+^ T cells (%)	1.103 (1.033–1.178)	0.003
	NK cells (%)	1.671 (1.191–2.345)	0.003
	CD4^+^ T/CD8^+^ T cells	5.480 (1.568–19.145)	0.008
W6	WBC (10^9^/L)	2.045 (1.249–3.348)	0.004
	CD8^+^ T cells (%)	0.839 (0.707–0.995)	0.044
	NK cells (%)	1.181 (1.002–1.394)	0.048
	CD4^+^ T/CD8^+^ T cells	8.512 (1.660–43.638)	0.010
ΔW3	CD4^+^ T cells (%)	1.022 (1.001–1.044)	0.043
	NK cells (%)	1.045 (1.016–1.075)	0.002
ΔW6	WBC (10^9^/L)	1.028 (1.004–1.052)	0.023
	CD8^+^ T cells (%)	0.920 (0.857–0.989)	0.023

**Table 3b:** Multivariate logistic regression of lymphocyte subpopulations percentage to predict objective response.

Variables		Odds ratio (95% Confidence Interval)	*p* value
W3	CD4^+^ T cells (%)	1.140 (1.020–1.274)	0.021
	NK cells (%)	2.232 (1.025–4.860)	0.043
ΔW3	NK cells (%)	1.035 (0.996–1.075)	0.078

W0, pretreatment; W3, 3 weeks post-treatment; W6, 6 weeks post-treatment; ΔW3=(W3-W0)/W0*100%; ΔW6=(W6-W0)/W0*100%.

**Figure 1:**
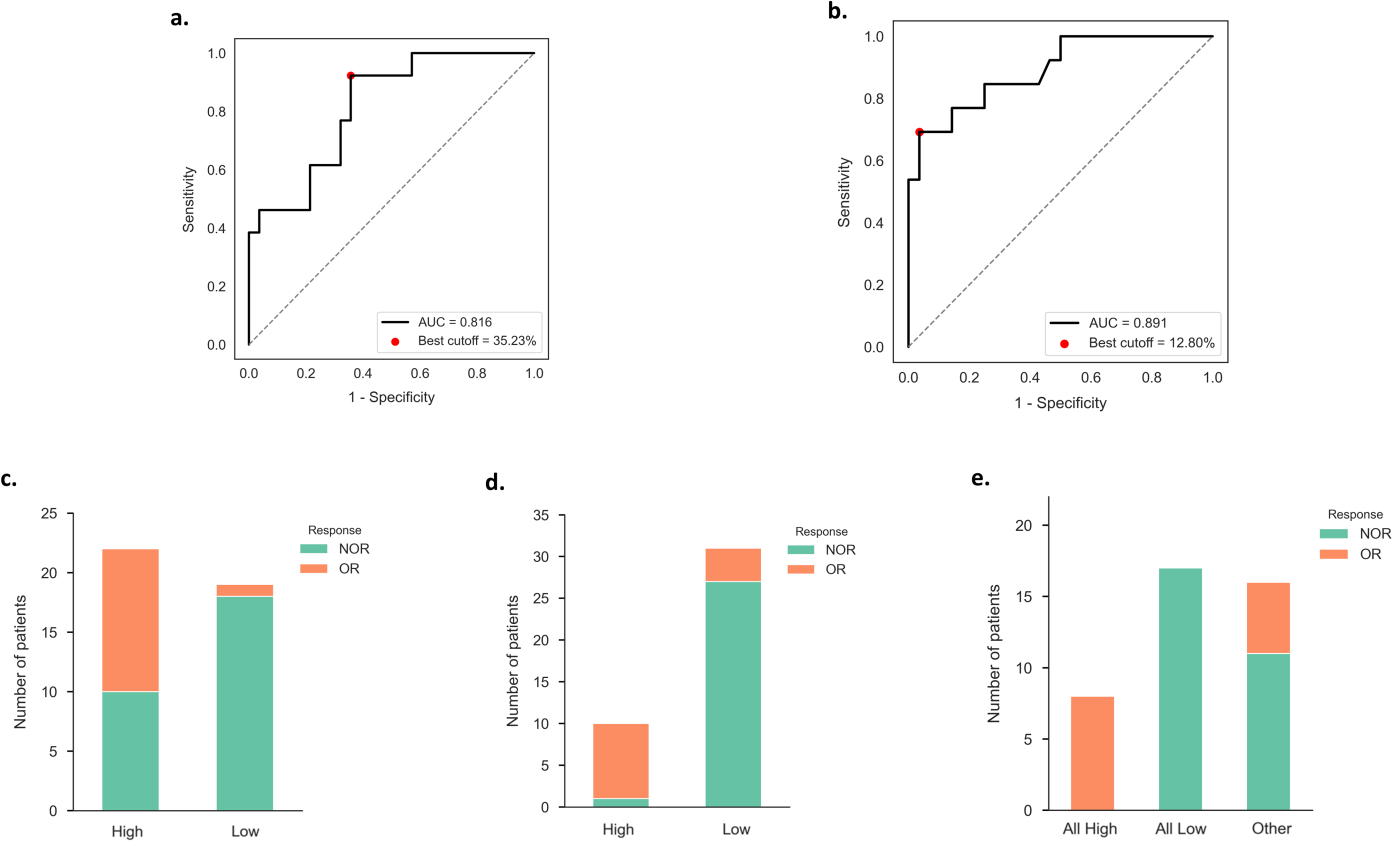
**(a)** ROC curve of W3 CD4^+^ T cells (%) for predicting OR. **(b)** ROC curve of W3 NK cells (%) for predicting OR. **(c)** Distribution of OR and NOR patients in the high or low W3 CD4^+^ T cells (%) groups (threshold: 35.23%). **(d)** Distribution of OR and NOR patients in the high or low W3 NK cells (%) groups (threshold: 12.80%). **(e)** Prediction of OR based on the combined two indicators: W3 CD4^+^ T cells (%) and W3 NK cells (%). W3, 3 weeks post-treatment; ROC, Receiver Operating Characteristic; AUC, Area Under the Curve; OR, Objective Response; NOR, Non-Objective Response.

To further evaluate their predictive performance, the ROC curves were generated, and the area under the curve (AUC) was calculated (**Figures 1a, b**). The AUC for CD4^+^ T cells (%) at W3 was 0.816, with the optimal cut-off value of 35.23%. For NK cells (%) at W3, the AUC was 0.891, and the optimal cut-off value was 12.80%. According to these cut-off values, the sensitivity and specificity were assessed. CD4^+^ T cells (%) at W3 showed a sensitivity of 92.3% and specificity of 64.3% (**Figure 1c**), whereas NK cells (%) at W3 exhibited a sensitivity of 69.2% and specificity of 96.4% (**Figure 1d**).

We observed that CD4^+^ T cells (%) at W3 yielded exceptionally high sensitivity, while NK cells (%) at W3 exhibited remarkably high specificity. Therefore, we combined these two markers to stratify patients into three groups: those with both values above the respective cut-off values were classified as the “All High” group, those with both values below the cut-off values as the “All Low” group, and the others as “Other” group. Remarkably, all patients in the “All High” group were classified as OR, whereas all patients in the “All Low” group were classified as NOR (**Figure 1e**), highlighting the strong predictive power by combining these two indicators.

**Figure 2:**
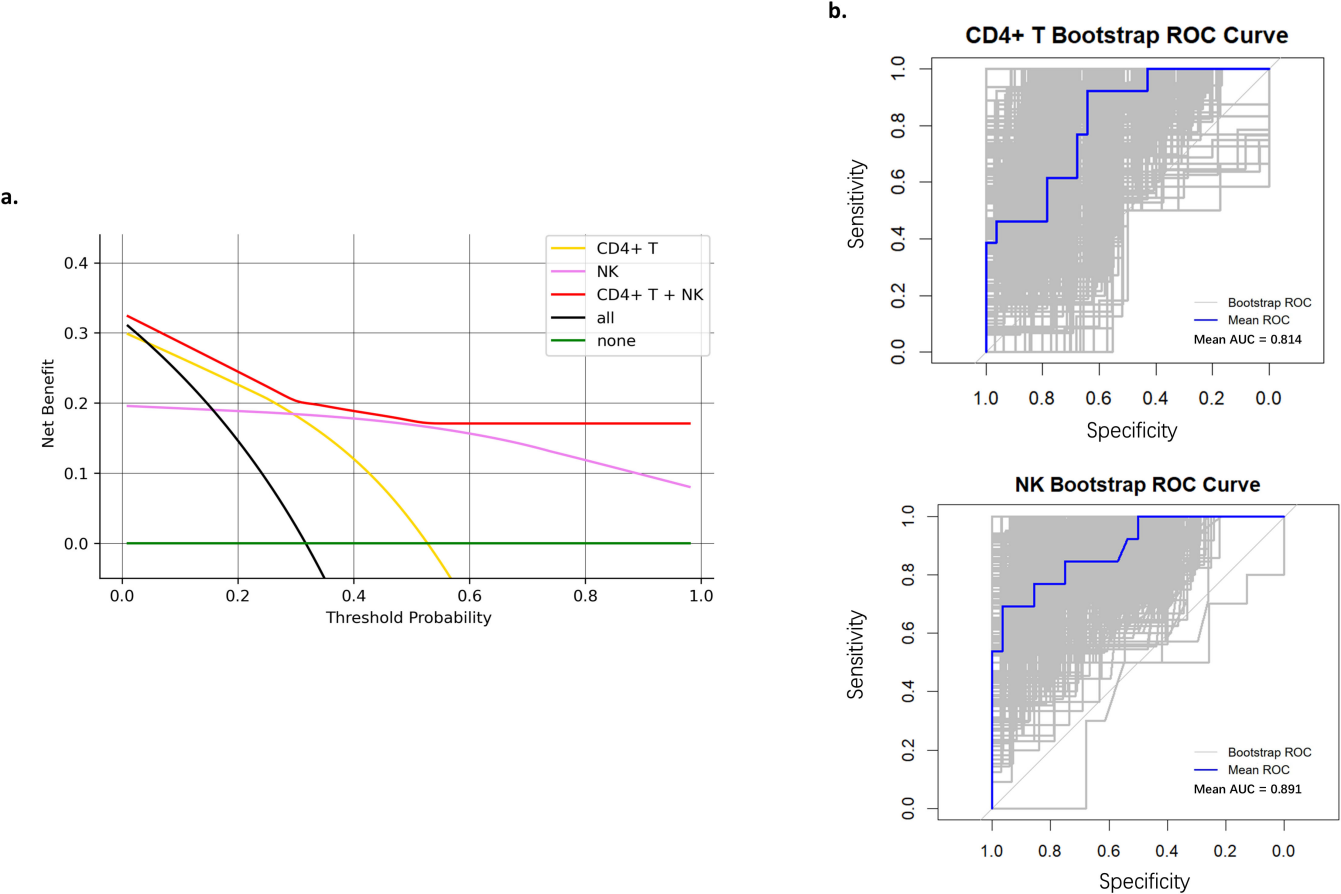
**(a)** The DCA curve of different ways to predict OR. **(b)** Bootstrap internal validation of W3 CD4^+^ T cells (%) and W3 NK cells (%). DCA, Decision Curve Analysis; OR, Objective Response; ROC, Receiver Operating Characteristic; AUC, Area Under the Curve.

**Figure 2a** shows the net benefit of different ways to predict OR. Using W3 CD4^+^ T (%) or W3 NK cells (%) adds more benefit than the treat-all or treat-non group. In addition, their combination showed a better net benefit than those alone. **Figure 2b** shows bootstrap internal validation of W3 CD4^+^ T (%) and W3 NK cells (%). The mean AUC is 0.814 and 0.891, respectively.

### 3.4 Analysis of OS and PFS

Patients were stratified based on OR or NOR, CD4^+^ T cells (%) at W3, (above or below 35.23%), and NK cells (%) at W3 (above or below 12.80%). Kaplan-Meier survival curves with log-rank tests were used to compare the differences in PFS and OS.

**Figure 3:**
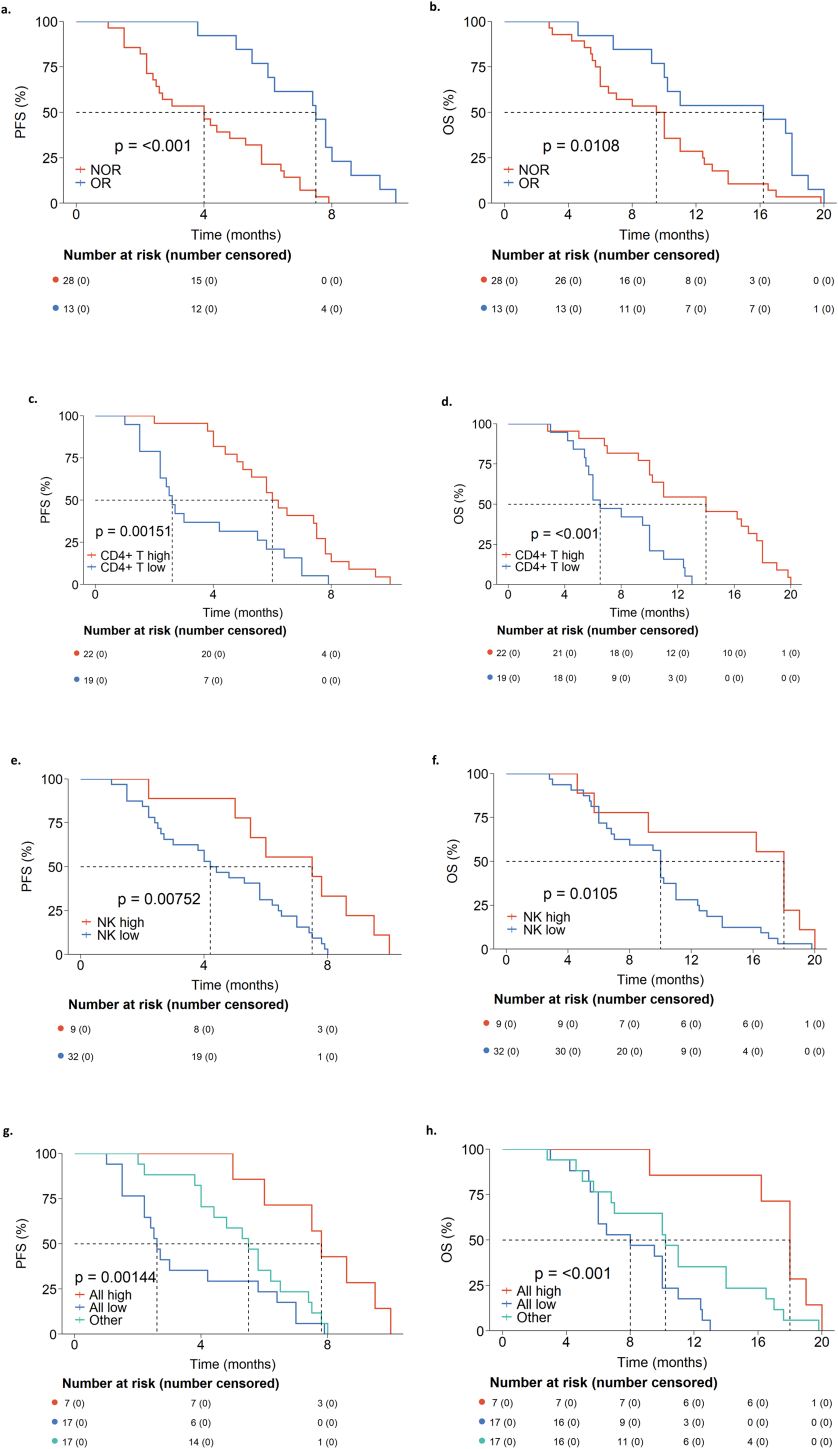
**(a)** Kaplan-Meier survival curve for PFS comparing OR and NOR groups. **(b)** Kaplan-Meier survival curve for OS comparing OR and NOR groups. **(c)** Kaplan-Meier survival curve for PFS comparing W3 CD4^+^ T cells (%) high and low groups. **(d)** Kaplan-Meier survival curve for OS comparing W3 CD4^+^ T cells (%) high and low groups. **(e)** Kaplan-Meier survival curve for PFS comparing W3 NK cells (%) high and low groups. **(f)** Kaplan-Meier survival curve for OS comparing W3 NK cells (%) high and low groups. **(g)** Kaplan-Meier survival curve for PFS across subgroups stratified by combined W3 CD4^+^ T cells (%) and W3 NK cells (%). **(h)** Kaplan-Meier survival curve for OS across subgroups stratified by combined W3 CD4^+^ T cells (%) and W3 NK cells (%). OR, Objective Response; NOR, Non-Objective Response; PFS, Progression-Free Survival; OS, Overall Survival; W3, 3 weeks post-treatment.

Patients in the OR group exhibited significantly longer PFS (median PFS: 7.5 months *vs*. 4.0 months; Hazard Ratio (HR) [95% CI]: 0.24 [0.11-0.53]; p ≤ 0.001; **Figure 3a**) and OS (median OS: 16.2 months *vs*. 9.5 months; HR [95% CI]: 0.41 [0.20-0.84]; p = 0.0108; **Figure 3b**) compared to the NOR group.

Patients with high CD4^+^ T cell (%) at W3 had longer PFS (median PFS: 6.0 months *vs*. 2.6 months; HR [95% CI]: 0.36 [0.18-0.69]; p = 0.00151; **Figure 3c**) and OS (median OS: 14.0 months *vs*. 6.5 months; HR [95% CI]: 0.22 [0.10-0.49]; p < 0.001, **Figure 3d**). Similarly, patients with high NK cell (%) at W3 also showed longer PFS (median PFS: 7.5 months *vs*. 4.2 months; HR [95% CI]: 0.30 [0.13-0.69]; p = 0.00752; **Figure 3e**) and OS (median OS: 18.0 months *vs*. 10.0 months; HR [95% CI]: 0.41 [0.18-0.90]; p = 0.0105; **Figure 3f**).

Furthermore, combined CD4^+^ T cells (%) and NK cells (%) detected at W3 was analyzed. Both PFS and OS followed the same pattern: All High > Other > All Low (median PFS: 7.8 months *vs*. 5.3 months *vs*. 2.6 months; median OS: 18.0 months *vs*. 10.2 months *vs*. 8.0 months; p = 0.00144 and < 0.001, respectively; **Figures 3g, h**).

These findings suggest that both indicators possess strong prognostic value for PFS and OS. The combination of these two markers enables enhanced stratification of patients who are more likely to derive long-term benefit.

## 4 Discussion

The advancement of immunotherapy has fundamentally transformed the treatment landscape for patients with advanced HCC. It could significantly prolong both PFS and OS (17). However, due to the heterogeneity, responses to immunotherapy vary considerably among patients. Our study demonstrated that patients in the OR group had significantly longer PFS and OS compared to those in the NOR group. Furthermore, we found that the percentages of W3 CD4^+^ T and W3 NK cells could predict the OR, and their combination yielded even better predictive performance. In addition, these two indicators, either individually or in combination, also demonstrated predictive value for PFS and OS in those patients.

Previous studies have explored other biomarkers, including the peripheral blood neutrophil-to-lymphocyte ratio (NLR) and circulating tumor DNA (ctDNA). Using the AUC as an indicator of predictive performance, the W3 CD4^+^ T cells (%) and W3 NK cells (%) identified in this study demonstrated superior predictive power (0.816 and 0.891, respectively) compared to previously reported biomarker (0.83 (18) for NLR and 0.852 (19) for ctDNA). These findings highlight the unique advantages of the W3 indicators identified in this study as a “minimally invasive, low-cost, and highly specific” biomarker.

Peripheral blood lymphocytes are one kind of white blood cell circulating in the bloodstream, comprising T cells, B cells, and NK cells (20). They are key components of immune system and play essential roles in adaptive immune responses and anti-tumor immunity, which are closely associated with immunotherapy response and long-term survival in cancer patients. Previous studies have demonstrated that in patients with advanced HCC subjected to immunotherapy, baseline levels of T and NK cells, along with an increase in NK cells following treatment, can predict therapeutic efficacy and long-term survival (10–12). In this study, we systematically evaluated the predictive value of the percentages of various lymphocyte subpopulations (CD4^+^ T cells, CD8^+^ T cells, and NK cells) at different time points (W0/baseline, W3, and W6), as well as their rate of change relative to baseline. The results revealed that high percentages of W3 CD4^+^ T cells (%) and W3 NK cells (%) were predictive of treatment efficacy and long-term survival (PFS and OS) in those patients. W3 represents a critical time point with profound biological implications. For immunotherapy to exert its effects, it must first reverse the suppression; these reactivated cells then undergo clonal expansion, differentiation, and migration to tumor sites to mount an anti-tumor immune response. W3 coincides with the convergence of these processes (21). Moreover, previous studies have shown that this time point aligns with Th1 cell differentiation and proliferation (22), as well as NK cell activation (23). Furthermore, there is evidence of interaction and mutual enhancement between these two cells (24). Potential immune mechanisms may include CD4^+^ T cells activating NK cells by secreting IFN-γ (25), and NK cells releasing antigens to enhance CD4^+^ T cells by killing tumor cells (26).

CD4^+^ T cells not only provide assistance to CD8^+^ T cells in killing tumor cells, but also directly eliminate tumor cells that express MHC class II molecules (27), or exert indirect anti-tumor effects by secreting cytokines that could activate other immune cells, such as macrophages (24, 28, 29). They play a central role in anti-tumor immune response. However, different subpopulations of CD4^+^ T cells, such as Th1, Th2, and Th17, are highly plastic within the tumor microenvironment and may exert opposing effects, either promoting or suppressing anti-tumor responses (30, 31). Although our study found that higher W3 CD4^+^ T cells (%) were associated with better treatment response and longer survival (PFS and OS), further research is still needed to determine which subpopulation of CD4^+^ T cell contributes to this finding. NK cells are so named because of their ability to kill tumor cells without prior sensitization (32, 33). As the key effector cells of the innate immune system and the first line of defense against cancer, NK cells play a critical role in tumor surveillance. Previous studies have shown that high levels of peripheral NK cells are associated with better treatment response and improved long-term survival in patients with advanced non-small cell lung cancer undergoing immunotherapy (34, 35), which is consistent with the findings of our study. These results further highlight the pivotal role of NK cells in anti-tumor immunity.

The limitation of our study is a single center, retrospective analysis with relatively small sample size, which may introduce selection bias and potential confounding factors. Meanwhile, the HBsAg positivity rate of patients in our center is relatively high, which was related to the local prevalence of hepatitis B (36). In future multi-center studies, HCC patients with different etiologies (such as hepatitis C, non-alcoholic fatty liver disease) will be included to verify the predictive efficacy of the indicators in different etiological subgroups. In addition, our analysis focused solely on CD4^+^ T cells, CD8^+^ T cells, and NK cells among peripheral blood lymphocytes. Whether more refined subpopulations such as regulatory T cells (Tregs), γδT cells, which are abundant in liver, or other types, like B cells, possess comparable or even superior predictive values remains to be explored in future research. Lastly, due to the limited sample size, subgroup analysis of different type of PD-1 inhibitors was not conducted, and future large-sample studies will explore the regulatory role of drug types.

## 5 Conclusion

Peripheral blood CD4^+^ T cells (%) and NK cells (%) measured at week 3 post-treatment serve as accurate and convenient biomarkers for predicting treatment efficacy and long-term outcomes (PFS and OS) in advanced HCC patients undergoing ICI immunotherapy. Their combination offers improved predictive performance.

## Data Availability

The datasets presented in this article are not readily available because of the policy of the Institutional Review Board of China-Japan Union Hospital of Jilin University. Requests to access the datasets should be directed to corresponding author.
