# Supplementary material for: Predictive value of peripheral blood CD4+ T and NK cells on efficacy and long-term survival in advanced HCC patients receiving immunotherapy
Source: Front Immunol. 2025 Nov 28;16:1683328. doi: 10.3389/fimmu.2025.1683328 (PMC12698629; doi:10.3389/fimmu.2025.1683328)
Supplement: Supplementary file 1 [file Table1.docx]

Table S1. Clinical characteristics of all patients regarding lymphocyte absolute count

| Variables | | n=41 |
| --- | --- | --- |
| W0 | CD4^+^ T cells (10^9^/L)  CD8^+^ T cells (10^9^/L)  NK cells (10^9^/L) | 0.53 (0.39, 0.74)  0.38 (0.32, 0.49)  0.19 ± 0.09 |
| W3 | CD4^+^ T cells (10^9^/L)  CD8^+^ T cells (10^9^/L)  NK cells (10^9^/L) | 0.72 (0.50, 0.94)  0.49 ± 0.16  0.19 (0.13, 0.23) |
| W6 | CD4^+^ T cells (10^9^/L)  CD8^+^ T cells (10^9^/L)  NK cells (10^9^/L) | 0.63 (0.52, 0.95)  0.45 (0.39, 0.54)  0.19 (0.15, 0.27) |

Continuous variables were expressed as mean ± standard deviation if normally distributed, or as median (interquartile range) if not. Categorical variables were presented as counts and percentages.

Abbreviations: W3 = 3 weeks post-treatment; W6 = 6 weeks post-treatment; NK = natural killer.

Table S2. Clinical characteristics regarding lymphocytes absolute counts comparison between objective response (OR) and non-objective response (NOR) patients

| Variables | | OR (13) | NOR (28) | p value |
| --- | --- | --- | --- | --- |
| W0 | CD4^+^ T cells (10^9^/L) | 0.62 (0.58-0.94) | 0.55 ± 0.23 | 0.166 |
|  | CD8^+^ T cells (10^9^/L) | 0.42 ± 0.09 | 0.36 (0.29, 0.44) | 0.183 |
|  | NK cells (10^9^/L) | 0.23 ± 0.09 | 0.17 ± 0.09 | 0.101 |
| W3 | CD4^+^ T cells (10^9^/L) | 1.04 (0.59-1.63) | 0.65 ± 0.23 | 0.107 |
|  | CD8^+^ T cells (10^9^/L) | 0.49 ± 0.15 | 0.49 ± 0.16 | 0.916 |
|  | NK cells (10^9^/L) | 0.28 (0.18-0.52) | 0.16 ± 0.09 | 0.141 |
| W6 | CD4^+^ T cells (10^9^/L) | 0.86 ± 0.35 | 0.62 (0.51, 0.80) | 0.107 |
|  | CD8^+^ T cells (10^9^/L) | 0.42 ± 0.10 | 0.49 ± 0.17 | 0.120 |
|  | NK cells (10^9^/L) | 0.24 (0.21-0.27) | 0.19 ± 0.08 | 0.149 |
| ΔW3 (%) | CD4^+^ T cells (10^9^/L) | 51.43 ± 59.17 | 32.97 ± 52.18 | 0.346 |
|  | CD8^+^ T cells (10^9^/L) | 18.94 ± 27.17 | 33.04 ± 43.79 | 0.216 |
|  | NK cells (10^9^/L) | 5.26 (-10.74-50.69) | 15.57 ± 61.55 | 0.705 |
| ΔW6 (%) | CD4^+^ T cells (10^9^/L) | 19.55 ± 31.78 | 39.42 ± 59.02 | 0.170 |
|  | CD8^+^ T cells (10^9^/L) | 4.44 (-2.75-17.70) | -0.21 (-12.22-83.12) | 0.685 |
|  | NK cells (10^9^/L) | 24.57 ± 52.97 | 12.57 (-8.72, 66.73) | 0.790 |
| ΔW3 (%)/ΔW6 (%) | CD4^+^ T cells (10^9^/L) | 1.56 ± 3.23 | 0.59 (0.15, 1.24) | 0.134 |
|  | CD8^+^ T cells (10^9^/L) | 0.38 ± 4.21 | 0.68 (0.31, 1.62) | 0.790 |
|  | NK cells (10^9^/L) | 0.45 (-0.21, 1.08) | 0.66 (0.03, 1.33) | 0.900 |

Continuous variables were expressed as mean ± standard deviation if normally distributed, or as median (interquartile range) if not. Categorical variables were presented as counts and percentages.

Abbreviations: W3 = 3 weeks post-treatment; W6 = 6 weeks post-treatment; NK = natural killer.
